# Khat *(Catha edulis)*-induced apoptosis is inhibited by antagonists of caspase-1 and -8 in human leukaemia cells

**DOI:** 10.1038/sj.bjc.6602197

**Published:** 2004-10-12

**Authors:** E A O Dimba, B T Gjertsen, T Bredholt, K O Fossan, D E Costea, G W Francis, A C Johannessen, O K Vintermyr

**Affiliations:** 1 Department of Odontology – Oral Pathology and Forensic Odontology, Faculty of Dentistry and Centre for International Health, University of Bergen, Bergen, Norway; 2 Hematology Section, Institute of Medicine, University of Bergen, Bergen, Norway; 3 Laboratory for Clinical Biochemistry, Haukeland University Hospital, Bergen, Norway; 4 Department of Chemistry, University of Bergen, Bergen, Norway; 5 Department of Odontology – Oral Pathology and Forensic Odontology, University of Bergen, Bergen, Norway; 6 Department of Pathology, The Gade Institute, Haukeland University Hospital, N-5021 Bergen, Norway

**Keywords:** Khat, leukaemia cells, apoptosis, caspases, cathinone, cathine

## Abstract

Khat chewing is a widespread habit that has a deep-rooted sociocultural tradition in Africa and the Middle East. The biological effects of khat are inadequately investigated and controversial. For the first time, we show that an organic extract of khat induces a selective type of cell death having all morphological and biochemical features of apoptotic cell death. Khat extract was shown to contain the major alkaloid compounds cathinone and cathine. The compounds alone and in combination also induced apoptosis. Khat-induced apoptosis occurred synchronously in various human cell lines (HL-60, NB4, Jurkat) within 8 h of exposure. It was partially reversed after removal of khat and the effect was dependent on *de novo* protein synthesis, as demonstrated by cotreatment with cycloheximide. The cell death was blocked by the pan-caspase inhibitor Z-VAD-fmk, and also by submicromolar concentrations of Z-YVAD-fmk and Z-IETD-fmk, inhibitors of caspase-1 and -8, respectively. The 50% inhibition constant (IC_50_) for khat (200 *μ*g ml^−1^)-induced apoptosis by Z-VAD-fmk, Z-YVAD-fmk and Z-IETD-fmk was 8 × 10^−7^ M as compared to 2 × 10^−8^ M and 8 × 10^−8^ M, respectively. Western blot analysis showed a specific cleavage of procaspase-3 in apoptotic cells, which was inhibited by Z-VAD-fmk. The cell death by khat was more sensitively induced in leukaemia cell lines than in human peripheral blood leukocytes. It is concluded that khat induces a rather swift and sensitive cell death by apoptosis through mechanisms involving activation of caspase-1, -3 and -8.

*Catha edulis* (khat) is a drug that is used by millions of people worldwide, mainly in Africa and the Middle East, for its psychostimulatory effects. The alkaloid fraction of khat is very efficiently extracted by chewing, and the major compounds are absorbed in the oral cavity (Toennes *et al*, 2003). Despite the body of knowledge on the adverse systemic effects of khat related to neurological disorders, hypertension, myocardial infarction and development of cancer (Al-Motarreb *et al*, 2002 for a recent review), so far, very little is known about the biological effects of khat on cells (Carvalho, 2003). Khat consumption leads to formation of micronuclei in human buccal and bladder mucosa, suggesting a genotoxic effect of khat use (Kassie *et al*, 2001). In rats, a decreased serum level of free radical metabolising/scavenging enzymes and glucose has been observed after oral administration, suggesting a deranged systemic capacity to handle oxidative radicals after khat use (Al-Qirim *et al*, 2002). It has also been reported that khat induces cytotoxic effects in cells (Al-Ahdal *et al*, 1988; Al-Meshal *et al*, 1991; Al-Mamary *et al*, 2002; Dimba *et al*, 2003) and in lymphoid tissue, and in the liver and kidney after per oral administration to white rabbits (Al-Meshal *et al*, 1991; Al-Mamary *et al*, 2002), but the mode of cell death has not so far been addressed.

Khat contains a number of pharmacologically active compounds (Kite *et al*, 2003). Cathinone is a major alkaloid component in fresh Catha leaves, but is relatively unstable and is rapidly metabolised into norpseudoephedrine (cathine) and norephedrine. Other alkaloid compounds such as the phenylpentenylamines and cathedulines could also contribute to pharmacological effects of khat (Kalix, 1992; Al-Motarreb *et al*, 2002). Most of their pharmacological effects are suggested as being mediated by release of biogenic amines through preferential binding to the norepinephrine receptor/transporter, but also partly through binding to dopamine and 5-hydroxytryptamine receptors (Rothman *et al*, 2003).

In the present study, we have tested an organic extract of khat on human leukaemia cell lines and primary peripheral leukocytes. We report that khat induces a swift and synchronised cell death having all the morphological and biochemical characteristics of apoptotic cell death. The cell death was dependent on *de novo* protein synthesis and more potent towards leukaemia cell lines than towards human peripheral blood leukocytes (PBLs). The khat alkaloids, cathinone and cathine, were also observed to induce apoptosis alone and more potently in combination. Induction of apoptosis by khat occurred through strictly regulated mechanism(s) that were sensitively regulated by cellular caspases.

## MATERIALS AND METHODS

### Materials

The caspase inhibitors Z-YVAD-fmk, Z-VDVAD-fmk, Z-DEVD-fmk, Z-WEHD-fmk, Z-VEID-fmk, Z-IETD-fmk, Z-LEHD-fmk and Z-VAD-fmk were from Medical and Biological Laboratories Co., Ltd. (Nagoya, Japan). Cathinone hydrochloride, cathine hydrocloride and bisbenzimide fluorochrome (Hoechst 33342) were from Sigma (St Louis, MO, USA). Jurkat and HL-60 cells were from the American Type Culture Collection (Manassas, VA, USA), while NB4 cells were a generous gift from Dr Michel Lanotte, L'Hopital Saint-Louis, Paris. Khat samples were from the Meru district in Kenya.

### Khat extraction

Fresh khat shoots, kept moist and transported at room temperature, were frozen 36–48 h after harvesting. Leaves were stored at −20°C for a maximum period of 5 days. The procedure for extraction of khat was a modification of the methanolic extraction protocol as previously described by Lee (1995), excluding alkaloid purification, so as to minimise acid or basic residues in the extract. The khat shoots (batches of 40 g) were swiftly chopped into small (5 mm) pieces and dissolved in 20 ml methanol. The mixture, shielded from light, was sonicated at RT for 15 min, and filtered through an 11 *μ*m filter (grade 1, Whatman, Kent, UK). The nonfiltered plant material was re-extracted in 20 ml fresh methanol and sonicated for 24 h. The mixture was filtered and admixed with the initial (15 min) methanol-extracted khat material. The resultant solution was then vacuum dried at 337 millibar in a Rotorvapor (Büchi, Switzerland) for 4–5 h into an oily paste. The dry weight of the extract was determined and thin layer chromatography was used to confirm the presence of alkaloids. Small aliquots of the extract were spotted directly onto a silica (Kieselgel F-254, Merck, Darmstadt, Germany) plate that was developed in ethylacetate :methanol : ammonia (85 : 10 : 5) followed by 0.5% ninhydrin solution and developed to detect the presence of cathinone and cathine (Zhou and Ninghua, 2000). In all, 40 g of fresh plant material yielded approximately 1.0 g of khat extract (range 0.9–1.2 g) and was dissolved in 5 ml DMSO. Aliquots (each 200 *μ*l) were immediately stored in −80°C and diluted in fresh medium prior to use. The different extraction batches were routinely tested and found to have similar effects suggesting that the isolation protocol was consistent in terms of preserved khat bioactivity.

### Analysis of khat by LC/MS/MS

Khat extract was diluted in methanol and water (5 : 95) before analysis. The concentrations of the khat calibration standards used were 0.01, 0.1, 1.0 and 10 *μ*g ml^−1^. Aliquots (2 *μ*l) were injected into the AB Sciex API 2000 LC/MS/MS system fitted with a TurboIonSpray interface (Applied Biosystems/Sciex, Toronto, Canada) and an Agilent 1100 series HPLC system (Agilent Technologies, Palo Alto, CA, USA). The analytes were separated (at 30°C) on a Betasil Phenyl column (50 × 2.1 mm i.d.: 3 *μ*m, Thermo Electron Corporation, Bellafonte, PA, USA) equipped with a Betasil Phenyl Javelin guard column (10 × 2.1 mm i.d.: 5 *μ*m, Thermo Electron Corporation, Bellafonte, PA, USA) and eluted from a separation gradient made from 0.1% formic acid (A) and acetonitrile (B) as follows: linear from 5–90% B; 0–5 min, 90% B; 5–6 min, linear from 90–5% B; 6–6.5 min, 5% B; 6.5–10 min. The flow rate was set to 0.30 ml min^−1^ during the separation procedure and positive electrospray ionisation was used in all experiments. To verify the presence of specific alkaloids in the khat extract, diluted samples (methanol : water; 5 : 95) of purified *S*(−)-cathinone hydrochloride, (1*S*,2*S*)-(+)-norpseudoephedrine (cathine) hydrochloride and DL-norephedrine hydrochloride (Sigma, St Louis, MO, USA) were used as standards.

**Figure 1:**
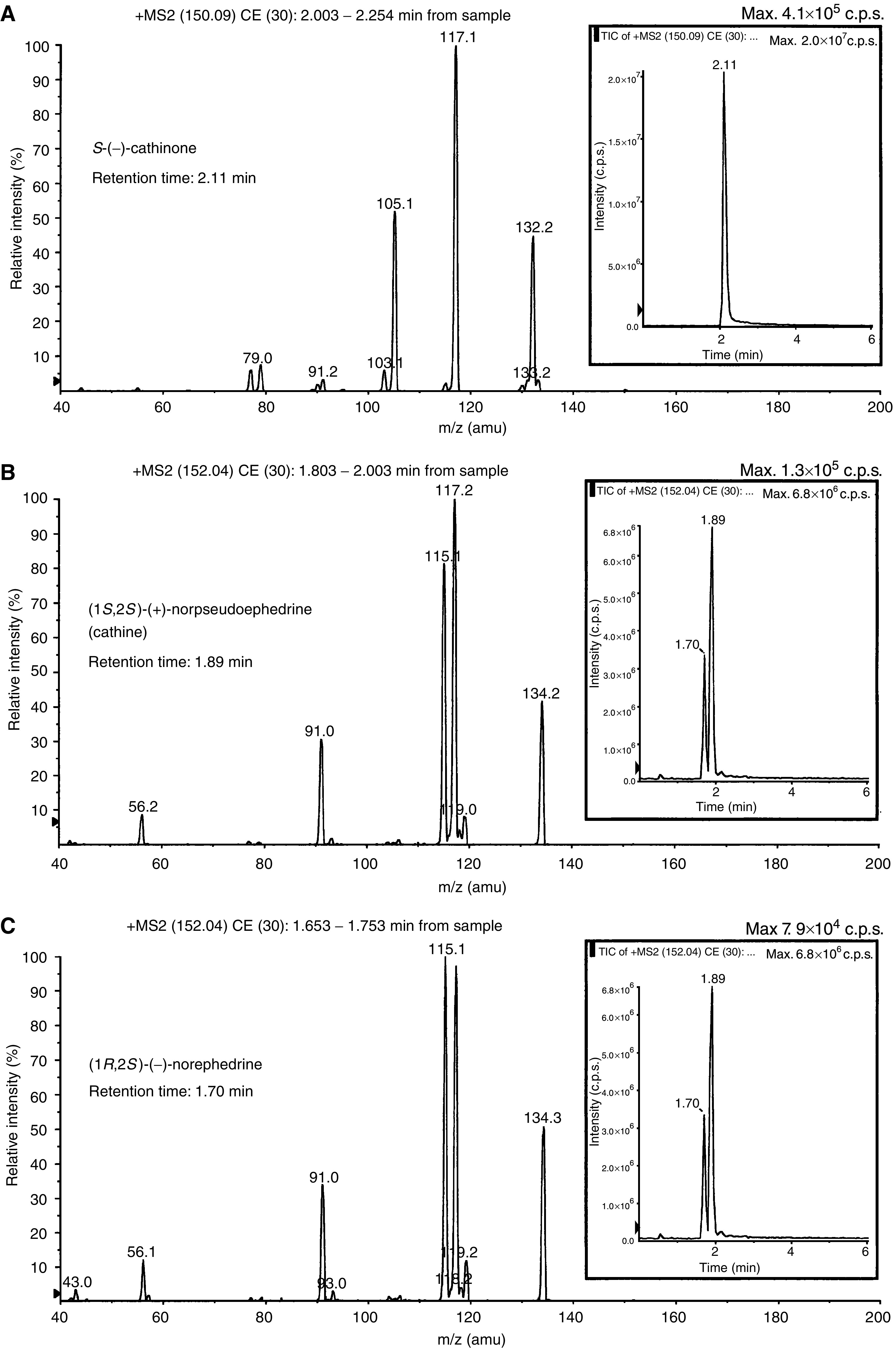
LC/MS/MS of the major khat alkaloids. Mass spectrometry analysis of diluted (1 : 2000) khat extract showing specific ion scan spectra of cathinone (panel **A** with precursor ion *m/z* 150), cathine (panel **B** with precursor ion *m/z* 152) and norephedrine (panel **C** with precursor ion *m/z* 152) run at collision energy of 30 eV. Inserts: Retention time determined by total ion chromatography from the diluted khat sample.

The phenylpropanolamine diastereomers cathine and norephedrine generated similar product ion scan mass spectra (Figure 1

). Quantitative analysis was performed in triplicate in the MRM mode, monitoring the following transitions: *m/z* 150 → 150, *m/z* 150 → 132 and *m/z* 150 → 117 for cathinone, and *m/z* 152 → 152, *m/z* 152 → 134 and *m/z* 152 → 117 for cathine and norephedrine. The concentrations of cathinone, cathine and norephedrine in the khat extract were 0.74±0.40, 1.49±0.51 and 0.9±0.16 mg ml^−1^ of khat, respectively.

### Cell culture and handling

HL-60, Jurkat and NB4 cells were cultured in RPMI 1640 medium (Sigma) supplemented with 10% heat-inactivated foetal bovine serum (Gibco, Grand Island, NY, USA), 2 mM L-glutamine (Gibco), 50 U ml^−1^ penicillin and 50 *μ*g ml^−1^ streptomycin (Gibco). Cells, in the early logarithmic growth phase, were seeded at a density of 2 × 10^5^ cells ml^−1^ in 24-well culture plates (Nunc, Roskilde, Denmark) 2 h before the start of experiments. The cultures were kept in a humidified atmosphere at 37°C, supplemented with 5% CO_2_. The concentration of DMSO was routinely kept below 0.1%. In experiments testing the effects of caspase inhibitors and cycloheximide, the cells were supplemented with these substances 15 min prior to the addition of khat.

In experiments investigating commitment to cell death after short-term khat exposure, 1 ml aliquots of treated cells were diluted in 10 vol. medium, and centrifuged (65 **g_av_**. for 5 min). The cell pellet was resuspended in 10 ml of complete medium, respun (65 **g_av_**. for 5 min), and the cells were admixed in 1 ml preconditioned complete culture medium. The preconditioned medium was obtained from parallel cultured normal (nontreated) HL-60 cells.

### Isolation of PBLs

In total, 10 ml of venous blood from each donor was diluted with 20 ml 0.9% NaCl, and 10 ml of Ficoll–Hypaque (NyCoMed, Oslo, Norway; specific density 1.077) was slowly pipetted into the mixture from the bottom of the tube. Samples were then centrifuged at 2000 r.p.m. for 25 min. The buffy coat (approximately 10 ml) was harvested, mixed with 4 vol. 0.9% NaCl and centrifuged at 1800 r.p.m. for 7 min. The pellet, average 500 *μ*l, was dissolved in 40 ml 0.9% NaCl and centrifuged (1800 r.p.m. for 7 min). The pellet was redissolved in RPMI supplemented with 10% FBS. The cells were seeded at an average concentration of 2 × 10^5^ cells ml^−1^ prior to addition of khat. This procedure gave an estimated dilution of patient serum proteins of more than 1 : 10 000 after the final suspension of human PBLs in FBS-supplemented RPMI medium (Bruserud *et al*, 2003).

### Determination of cell death

#### Dye exclusion test

The cell membrane integrity was assessed by the ability to exclude 0.2% trypan blue. The fraction of cells that excluded trypan blue was counted in a haemocytometer. In each determination, a minimum of 200 cells was evaluated. The fraction of viable cells was expressed as an arithmetic mean±standard error of the mean (s.e.m.).

#### Nuclear chromatin condensation test

Small (50 *μ*l) cell aliquots were added to 50 *μ*l fixative that contained 4% formaldehyde supplemented with 10 *μ*g ml^−1^ of the DNA-specific fluorochrome, bisbenzimide (Hoechst 33342; Sigma, St Louis, MO, USA) at various time points. Normal (nonapoptotic) cells had a uniform diffuse nuclear fluorescence. The morphologically altered cells (apoptotic cells) had more condensed and intensely stained nuclear features, often with typical margination of the nuclear chromatin, with or without fragmented nuclei. The fraction of apoptotic cells was determined in randomly selected areas containing approximately 100 cells (range 90–130) under epifluorescence microscopy using a Leica IRB inverse microscope with × 400 magnification essentially as previously described (Gjertsen *et al*, 1994; Jensen *et al*, 1994).

#### Determination of loss of microvilli

Quantification of loss of microvilli was carried out in bright field microscopy using a Leica IRB inverse microscope with × 400 magnification. Normal cells were characterised by abundant microvilli on the cell surface, while apoptotic cells had a smooth contour with loss of microvilli with very few cells in an intermediate phase. The fraction of apparent normal cells was determined in randomly selected areas containing approximately 100 cells (range 90–130).

#### Electron microscopy

Cells were fixed in 0.1 M Na-cacodylate buffer, pH 7.4. containing 2% glutaraldehyde. Samples were then rinsed three times with buffer and postfixed in 1% osmium tetroxide. The specimens were then dehydrated using graded ethanols and embedded in epoxy resin, and ultrathin sections double stained with uranyl acetate and lead citrate (Bøe *et al*, 1995). Specimens were examined with electron microscopy (JEOL-1230, Jeol Ltd., Tokyo, Japan). The micrographs were processed using an AGFA Arcus II scanner and Adobe Photoshop 6.0 software.

### Western blot analysis

The procedure for Western blot was mainly as previously described (Bruserud *et al*, 2003). At designated time points, 10 × 10^6^ cell samples were washed in ice-cold 0.9% NaCl and lysed in 10 mM Tris (pH 7.5), 1 mM EDTA, 400 mM NaCl, 10% glycerol, 0.5% NP-40 and 5 mM NaF supplemented with fresh DTT (1 mM), orthovanadate (1 mM) and Complete protease inhibitor (Roche Molecular Biochemicals, Oslo, Norway). Samples were homogenised with a minipiston on ice and centrifuged (13 000 **g_av_**., 15 min, 4°C). The free supernatant was carefully removed and stored in −80°C until further use. Aliquots with equal amounts of total cell proteins (determined using a Bio-Rad protein assay standard) were pretreated with 3 × SDS–PAGE buffer and resolved using 12.5% polyacrylamide SDS-denaturing gels. The proteins were then transferred onto polyvinylfluoride (PVDF) membranes (Hybond, Amersham Pharmacia Biotech, Little Chalfont, UK). Procaspase-3 and activated caspase-3 cleavage products were probed with *α* anti-caspase-3 E8 primary antibody (Santa Cruz Inc., Santa Cruz, CA, USA) followed by *α* anti-mouse-conjugated horseradish peroxidase (Jackson ImmunoResearch Laboratories, Inc., West Grove, PA, USA). An antibody against *β*-actin was used as an internal loading control. The immunoblot detection was performed by enhanced chemiluminescence (Pierce Biotechnology, Rockford, IL, USA) acquired on a KODAK Image Station 2000R.

### Flow cytometry

Dual colour flow cytometry with Annexin-V-FITC and propidium iodide (Nexin Research, Kattendijke, The Netherlands) was used to probe apoptotic and necrotic fractions in NB4 cells and in human donor-derived PBLs exposed to khat extracts for 8 h. Cells (1.0 × 10^6^) were labelled with Annexin V (125 ng sample^−1^) and propidium iodide (2.5 *μ*g ml^−1^) according to the procedure of the manufacturer. Similarly, the fluorescent probe, 5,5′,6,6′ tetrachloro-1, 1′,3,3′-tetraethylbenximidazol-carbocyanine iodide (JC-1; Molecular Probes Inc., Eugene, OR, USA) was used to probe mitochondrial membrane potential in cells undergoing khat-induced apoptosis. All flow cytometric analyses were carried on a FACScan machine (Becton Dickinson, Franklin Lakes, NJ, USA).

### Statistics

All data were expressed as the mean±s.e.m. To compare cell death between multiple treatment groups, SPSS version 11.0 was used to perform an ANOVA test followed by Wilks' Lambda *F* test to determine statistical significance (*P*<0.05).

## RESULTS

### Khat induced morphological effects resembling apoptotic cell death

**Figure 2:**
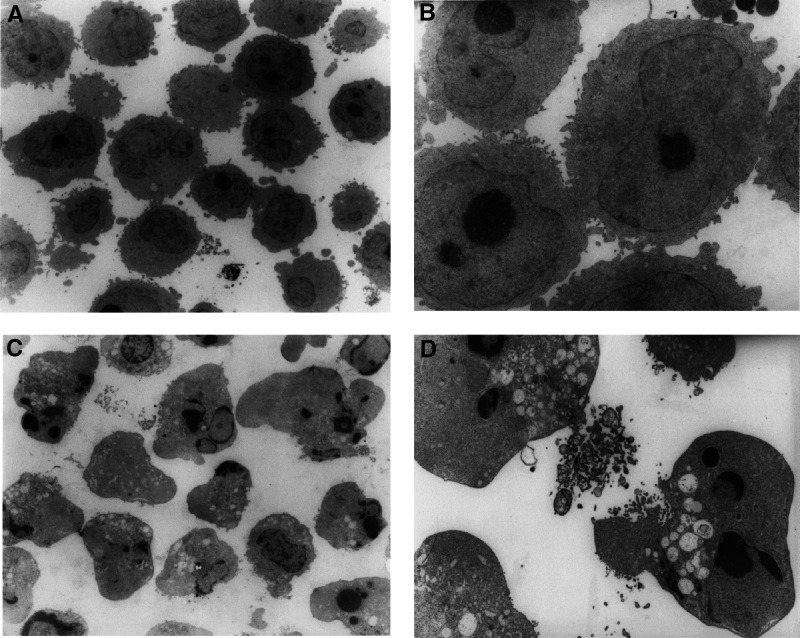
Morphological effects of khat in HL60 cells. HL-60 cells in early logarithmic growth phase were exposed to an organic extract of khat (200 *μ*g ml^−1^) for 8 h (panels **B** and **D**) or left nonsupplemented (control treated with DMSO solvent) (panels **A** and **C**). The cellular morphology was visualised by electron microscopy at × 1000 magnification (panels **A–B**) and at × 6000 magnification (panels **C**, **D**).

An organic extract of khat was found to induce profound morphological effects in HL-60 human acute myeloid leukaemia cells. These changes were characterised by an early loss of microvilli and by major cell conformational changes including blebs or buds on the cell surface membrane (Figure 2

). High-power electron microscopy revealed a series of morphological changes in the cells, including cell shrinkage, segregation of intracellular organelles, formation of membrane blebs some of which contained organelles, the appearance of vacuoles in the cytoplasm, disruption of the nuclear membrane, and condensation and fragmentation of nuclear chromatin (Figure 2C).

**Figure 3:**
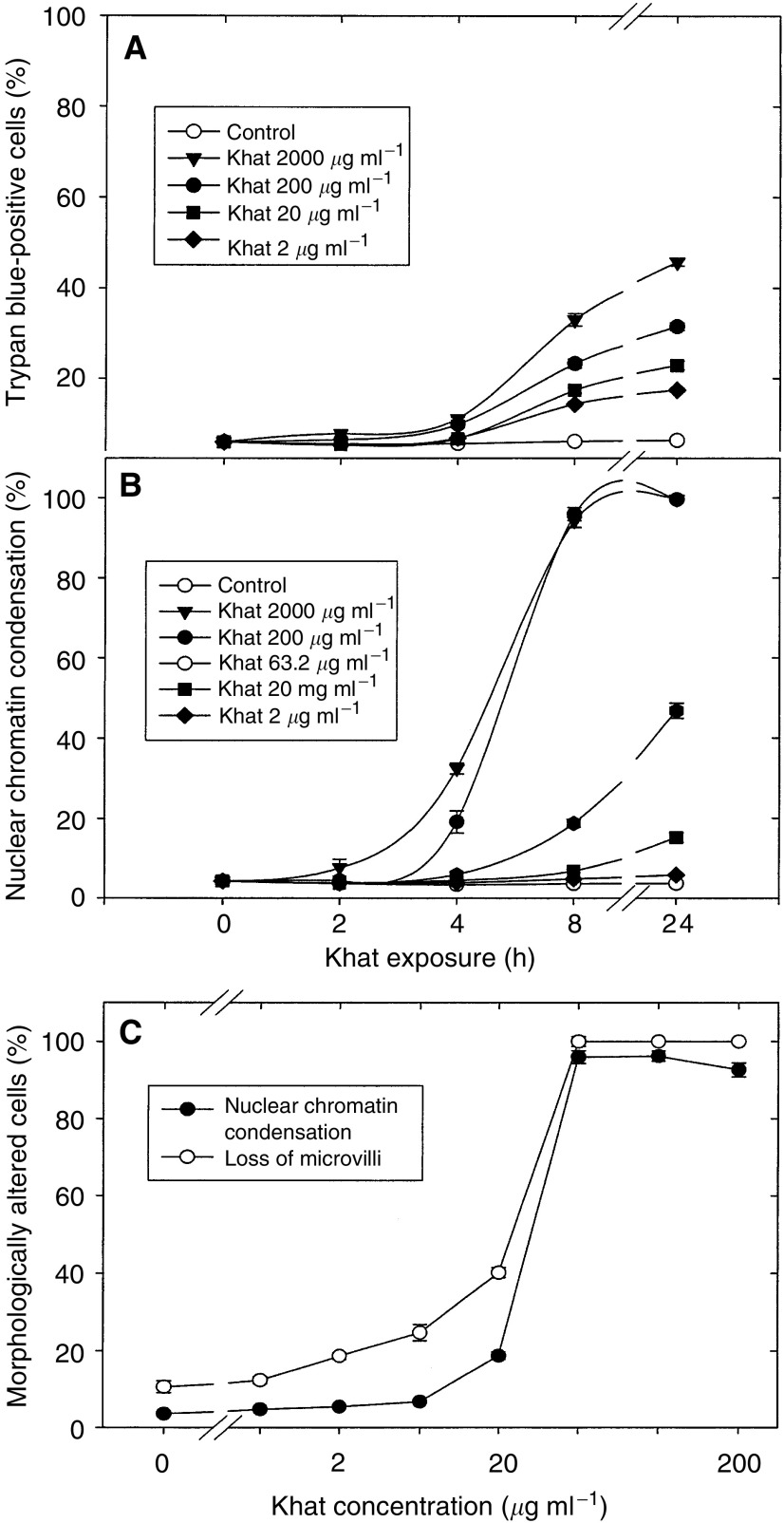
Effect of khat on cell permeability (dye exclusion test) nuclear chromatin condensation and loss of microvilli. (**A**, **B**) HL-60 cells were incubated with various concentrations of khat (range 2–2000 *μ*g ml^−1^) or left nonsupplemented as controls. At various time points, cell aliquots were tested for ability to exclude trypan blue (dye exclusion test) (**A**) or for condensation of nuclear chromatin (**B**). The data represent the mean±s.e.m. of three separate experiments each in triplicate.) Cells were exposed to various concentrations (range 2–200 *μ*g ml^−1^) of khat (•) or left nonsupplemented as controls (○). After exposure for 8 h, cell aliquots were fixed with formaldehyde and the fraction of cells with loss of microvilli determined. The data represent the mean±s.e.m. of three experiments in triplicate.

Khat-exposed cells were tested with respect to their ability to exclude trypan blue. Uptake of trypan blue in exposed cells was a relatively late event compared with the marked morphological changes associated with early khat exposure (Figure 3A

). Among cells exposed to 200 *μ*g ml^−1^ khat for 8 h, more than 85–90% of the cells showed condensation of nuclear chromatin, whereas more than 65% of the cells retained their ability to exclude trypan (Figure 3A and B). Khat induced nuclear chromatin condensation in a concentration-dependent manner at concentrations above 20 *μ*g ml^−1^ of extract. The effect had a swift onset and was significant after 2–4 h exposure time in a broad range of concentrations above 6.3 *μ*g ml^−1^ of khat.

The effect of khat was also tested by determination of loss of microvilli. Normal (nontreated) HL-60 cells had a preponderance of microvilli on the cell surface. After exposure to khat, loss of cell surface microvilli appeared to be a very sensitive and consistent morphological marker for khat-induced cell death. This effect closely mimicked the timing for onset of khat-induced condensation of nuclear chromatin, but was generally more sensitive than the latter parameter, especially at low khat concentrations (Figure 3C).

### Khat-induced cell death was characterised by activation of caspase-3

**Figure 4:**
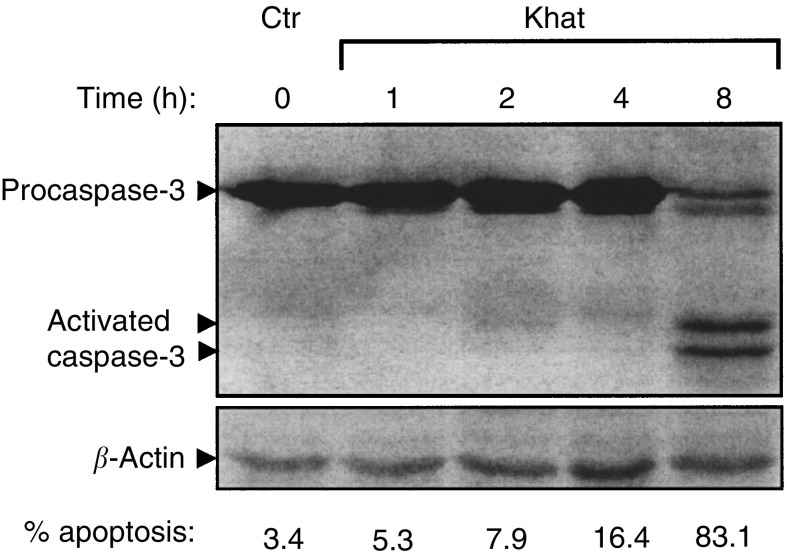
Activation of procaspase-3 in khat-exposed cells. Cell lysates were prepared and proteins separated by SDS–PAGE and transblotted onto PVDF membranes. Caspase-3 was probed by mouse *α*-caspase-3 and detected using a horseradish peroxidase-coupled secondary antibody (see Materials and Methods). *β*-Actin was used as an internal reference marker to monitor the amount of protein in each lane.

Activation of procaspase-3 by means of proteolytic cleavage is considered a major pathway in the execution phase of apoptosis (Herngartner, 2000). In a high-speed cytosolic fraction (see Materials and methods section for details), the 32 kDa procaspase-3 was selectively cleaved in khat-treated cells to 17 and 19 kDa fragments as visualised on immunoblotted SDS–PAGE gels (Figure 4

). The cleavage of procaspase-3 paralleled the morphological effects of apoptosis.

### Apoptotic cell death was induced by khat-specific phenylpropylamines

HL60 cells were exposed to cathinone and cathine singly or in combination (Table 1

**Table 1:** Induction of apoptosis in HL60 by khat-specific phenylpropylamines

**Alkaloid concentration (M)**	**Cathinone (% apoptosis mean±s.e.m.)**	**Cathine (% apoptosis mean±s.e.m.)**	**Cathinone+cathine (% apoptosis mean±s.e.m.)**
0	4.35±0.8024	4.23±0.9755	3.61±1.1842
10^−8^	5.72±0.8400	4.96±1.2594	22.31±1.1181
10^−7^	16.35±1.5820	12.44±0.8513	23.91±1.9656
10^−6^	24.45±1.2298	13.90±0.7776	24.37±2.2349
10^−5^	30.52±1.1510	23.67±2.5663	75.69±4.4470
10^−4^	44.76±0.9528	28.70±3.3373	85.19±3.6467

HL-60 cells in early logarithmic growth phase were exposed to various concentrations (column 1) of cathinone (column 2), cathine (column 3) or cathinone and cathine in combination (column 4). The fraction (%) of cells with condensed nuclear chromatin was determined after 8 h of exposure. Cells with condensed nuclear chromatin were considered apoptotic. The experiment was conducted twice each in triplicate. The mean±s.e.m. is shown for each concentration of substance tested.

). These compounds induced cell death within concentration ranges obtained in cells exposed to khat extract (see Materials and Methods section). Cathinone was somewhat more potent than cathine in causing cell death. Of particular interest was the observation that the effect of these compounds used in combination seemed to be more potent than when used separately. This apparent interaction emphasises the importance of testing whole khat extract as well as its purified constituents for a full investigation of biological effects.

### Cell death by khat in various human leukemia cell lines and in human PBLs

**Figure 5:**
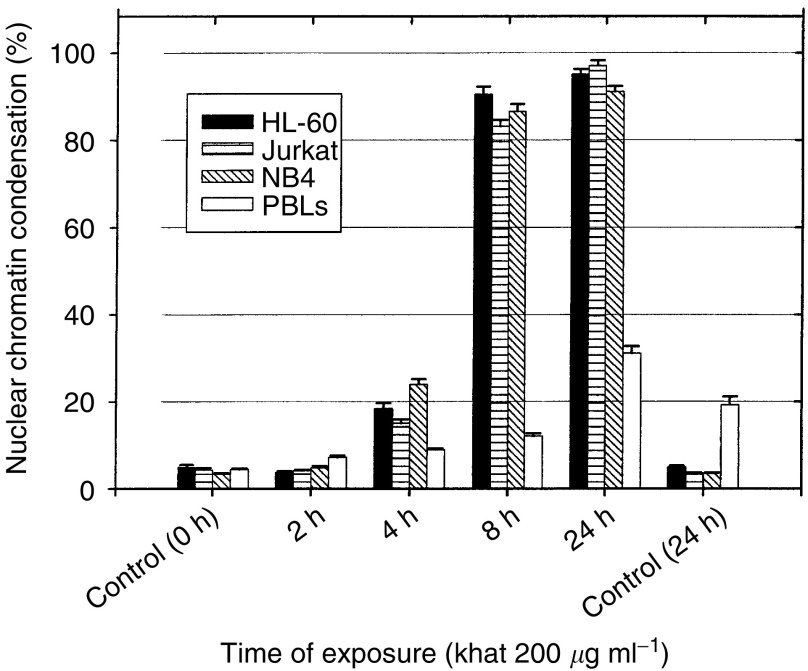
Human leukaemia cell lines undergo cell death (apoptosis) more sensitively than peripheral blood human leukocytes (PBLs) after exposure to khat. Various human acute myeloid (HL-60, NB4) and lymphoblastic leukaemic (Jurkat) cell lines as well as isolated PBLs were exposed to 200 *μ*g ml^−1^ khat for various time points or left nonsupplemented as controls. The fraction of cells with condensed nuclear chromatin was determined. The data on the cell lines represent the mean±s.e.m. of one experiment in triplicates, whereas the data on PBLs were obtained from three healthy individuals, each run in triplicates. The differences between khat-treated and untreated cells, and between khat-treated cell lines and PBLs were statistically significant (*P*<0.05).

The ability of khat to induce cell death was further tested in other leukaemic cell lines (NB4 and Jurkat cells) and in isolated PBLs. It was found that khat induced morphological effects of apoptosis in NB4 and in Jurkat cells and at potencies similar to those observed in HL-60 cells (Figure 5

). Khat also induced cell death in isolated PBL cells, but with less sensitivity (Figure 5). These cells were also analysed by flow cytometry using Annexin-V-FITC and propidium iodide after khat exposure and showed similar results as with the morphological studies (data not shown).

### The commitment to cell death by khat was partially reversible

**Figure 6:**
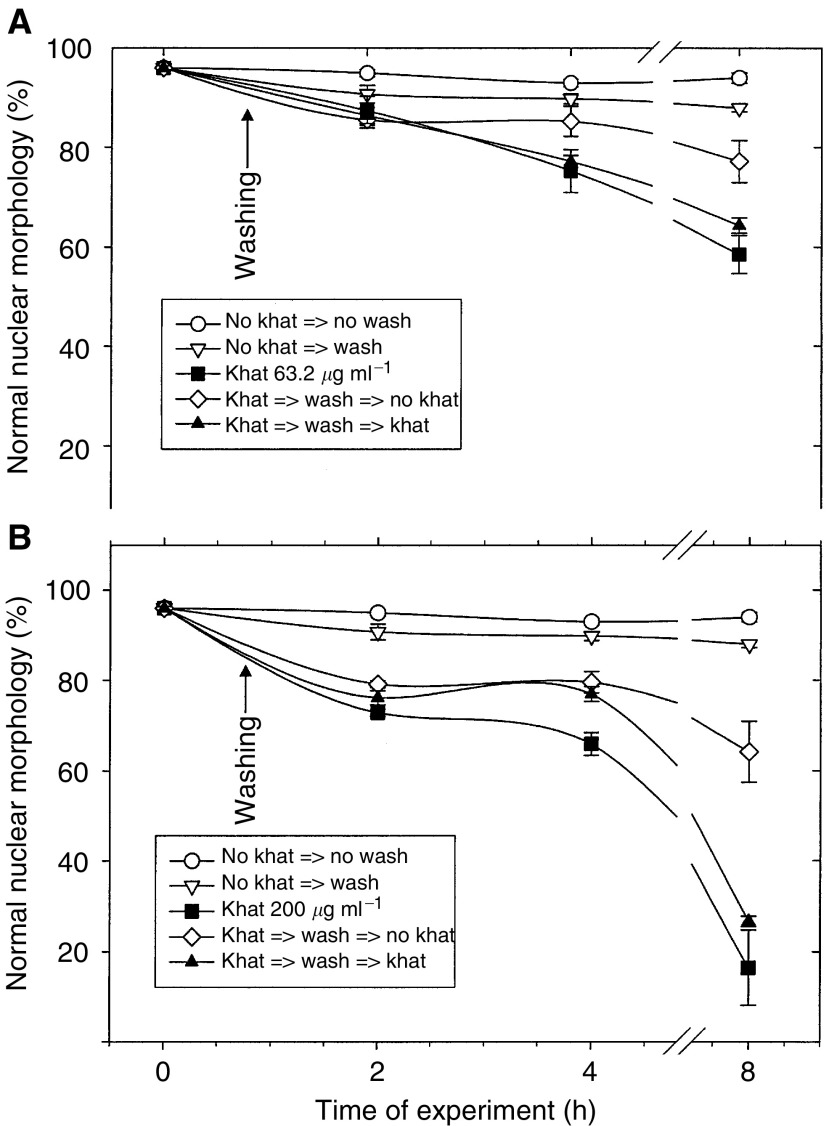
Test on commitment to cell death after short-term exposure to khat. HL-60 cells were exposed to 63.2 *μ*g ml^−1^ khat (**A**) or 200 *μ*g ml^−1^ khat (**B**) for 0.5 h, washed, and left nonsupplemented (open symbols) or resupplemented (filled symbols) with khat. Some cultures were treated continuously with 63.2 *μ*g ml^−1^ khat (▪, panel **A**) or 200 *μ*g ml^−1^ khat (▪, panel **B**) as reference. Other cultures were left unsupplemented with khat with (▿) or without washing (○). At the times indicated, cell aliquots (50 *μ*l) were mixed with one volume fixative. Cell survival (%) was based on determination of the fraction of cells not having condensed nuclear features. These morphological findings were considered typical of cells irreversibly committed to death. The data represent the mean±s.e.m. of triplicate experiments. The difference between khat-treated samples and untreated (controls) were statistically significant (*P*<0.05).

The duration of khat exposure needed to evoke cell death in HL-60 cells was then tested. The cells were exposed to 63.2 or 200 *μ*g ml^−1^ khat for short periods of time and then washed and replenished with conditioned medium. Cells with condensed nuclear chromatin were considered to be irreversibly committed to cell death, whereas cells with normal nuclear features were healthy (Jensen *et al*, 1999). In cells treated with 63.2 *μ*g ml^−1^ khat for 0.75 h, the effect of khat could be abolished (Figure 6A

), whereas in cells exposed for 1 or 2 h the effect of khat was only partially abolished (data not shown). In cells exposed to 200 *μ*g ml^−1^ khat for 0.75 h (Figure 6B), only a partial and temporary rescue was observed. Cells that were exposed to 63.2 and 200 *μ*g ml^−1^ khat for 2 h or more did not show partial or temporary rescue from cell death (data not shown). The above results showed that khat (63.2 *μ*g ml^−1^) had to be present for more than 0.5–0.75 h to induce cell death and that exposure time for more than 2 h had no cytopreventive effect as compared to continuously exposed cells.

### Cell death by khat was dependent on *de novo* protein synthesis

**Figure 7:**
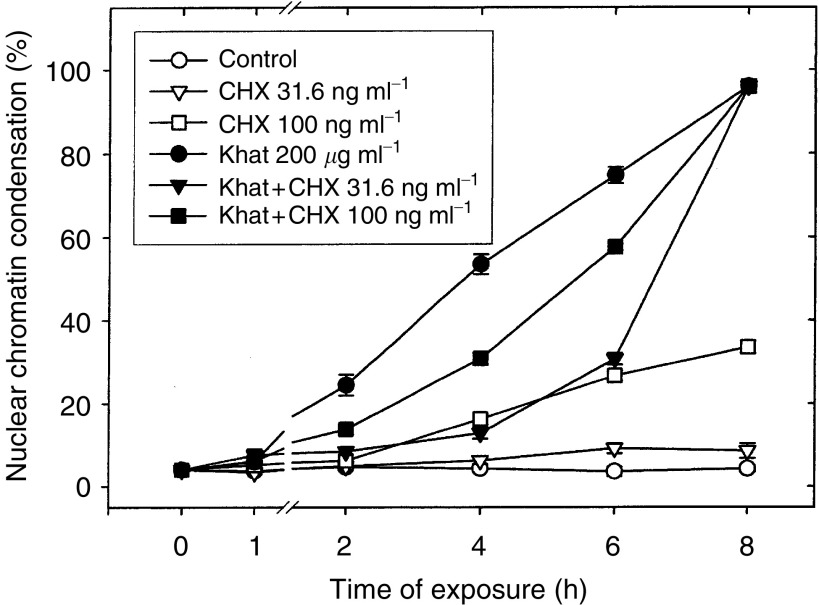
Khat-induced cell death in HL-60 cells was dependent on *de novo* protein synthesis. Cells were exposed to 200 *μ*g ml^−1^ khat in the absence (•) or presence of 31.6 ng ml^−1^ CHX (▾) or 100 ng ml^−1^ CHX (▪). Some cultures were left unsupplemented (○) as controls or treated with 31.6 ng ml^−1^ CHX (▿) or 100 ng ml^−1^ CHX (□). CHX was added 15 min prior to treatment with khat. At the times indicated, cell aliquots (50 *μ*l) were removed and dead cells determined as the fraction of cells (%) having condensed nuclear chromatin. The data represent means±s.e.m. of two separate experiments, each in triplicate.

To investigate whether cell death induced by khat was dependent on *de novo* protein synthesis, khat-exposed cells were cotreated with cycloheximide (CHX). First, the effect of various concentrations (range 31.6–1000 ng ml^−1^) of CHX was tested on cell survival. The lower concentrations of CHX only marginally (CHX 31.6 ng ml^−1^) or moderately (CHX 100 ng ml^−1^) affected cell death in exposed HL-60 cells (Figure 7

). The higher concentrations (CHX >316 ng ml^−1^) induced a rather pronounced cell death on its own (data not shown). In the presence of low to moderate CHX –concentrations, the cell death effect by khat was inhibited (Figure 7). In the time interval 2–6 h, CHX (31.6 ng ml^−1^) inhibited the cell death effect of 200 *μ*g ml^−1^ khat by 77% (range 67–82%) when corrected for spontaneous apoptosis and a slight independent cell death promoting effect of 31.6 ng ml^−1^ CHX. The effect was, however, temporary; that is, the morphological effect of khat was not inhibited by CHX in cells exposed to khat for longer (8 h or more) time periods (Figure 7).

### Antagonism of khat-induced cell death by caspase inhibitors

**Figure 8:**
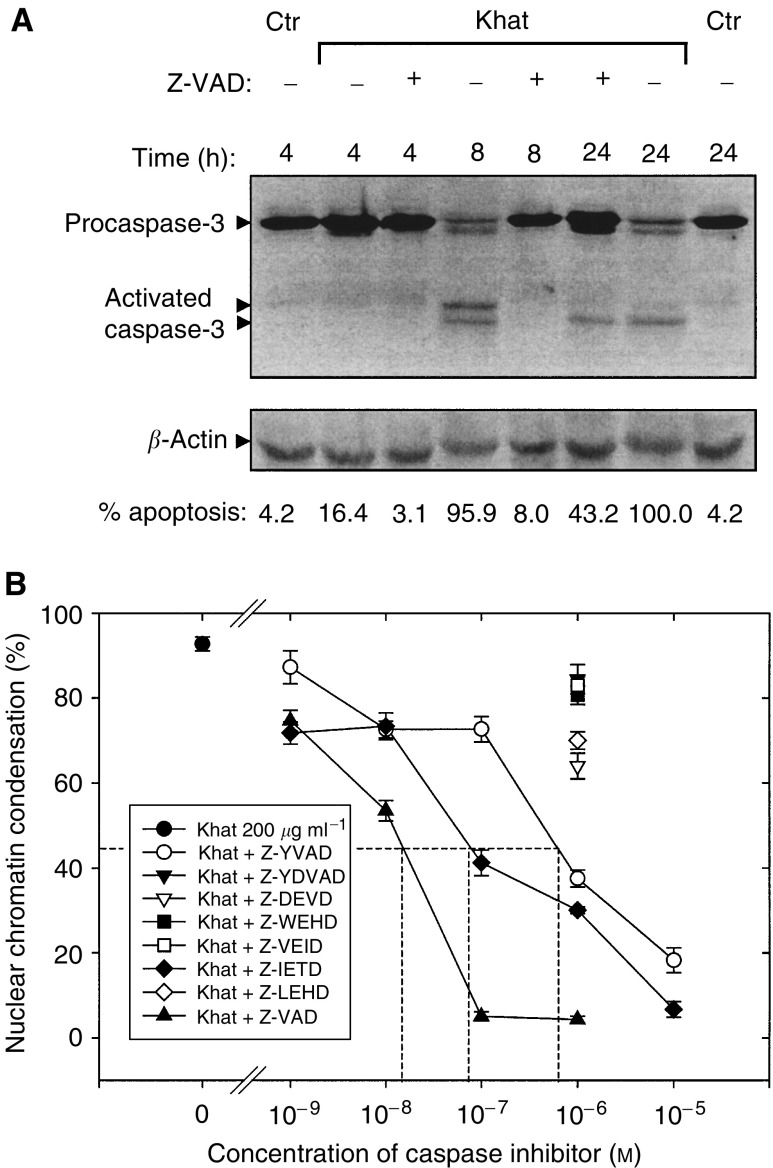
Selective inhibition of khat-induced cell death by caspase inhibitors. (**A**) Cultures of HL-60 (10 × 10^6^) cells were exposed with 200 *μ*g ml^−1^ khat for various time points in the absence or presence of 10^−6^ M Z-VAD-fmk or left nonsupplemented as control cultures. (**B**) HL-60 cells were treated with 200 *μ*g ml^−1^ khat for 8 h in the absence () or presence of various concentrations of Z-VAD-fmk (), a pan-caspase inhibitor, or the caspase selective inhibitors Z-YVAD-fmk () and Z-IETD-fmk (), inhibiting caspase-1 and -8, respectively. For the caspase inhibitors Z-VDVAD-fmk, Z-DEVD-fmk, Z-WEHD-fmk, Z-VEID-fmk and Z-LEHD-fmk, selecting predominantly inhibiting caspases-2, -3, -5, -6 and -9, respectively, only the effect of 10^−6^ M concentration of these inhibitors is shown. Half maximal inhibitory concentration (ID_50_) for induction of cell death by 200 *μ*g ml^−1^ khat extract after 8 h of exposure was determined to be 2 × 10^−8^, 9 × 10^−8^ and 8 × 10^−7^ M for Z-VAD, Z-IETD and Z-YVAD, respectively. The data represent means±s.e.m. of two separate experiments, each in triplicates.

In a new set of experiments, cells were exposed with khat in the presence or absence of Z-VAD-fmk. The pan-caspase inhibitor Z-VAD-fmk was found to be a very sensitive inhibitor of khat-induced cell death. The cleavage of procaspase-3 was inhibited by the presence of Z-VAD-fmk in the culture medium (Figure 8A

). Half-maximal inhibitory concentration (IC_50_) of Z-VAD-fmk was determined to be 2 × 10^−8^ M in cells exposed to 200 *μ*g ml^−1^ khat for 8 h (Figure 7B). A panel of caspase inhibitors with selectivity towards caspases 1–3, 5–6 and 8–9 was similarly used to investigate specific inhibitory effects. It appeared that Z-YVAD-fmk and Z-IETD-fmk, inhibiting caspase-1 and -8, respectively, were also effective, although with less potency than Z-VAD (Figure 8B). The caspase inhibitors Z-DEVD-fmk and Z-LEHD-fmk, inhibitors of caspase-3 and -9, respectively, had a slight but significant effect (Figure 7B), whereas Z-VDVAD-fmk, Z-WEHD-fmk and Z-WEID-fmk, inhibiting caspase-2, -5 and -6, respectively, had no significant effect on khat-induced cell death.

## DISCUSSION

In this study, we report that a standardised organic extract of khat induced a very consistent and reproducible type of cell death in various human leukemia cell lines and in peripheral human blood leukocytes (Figure 2). Khat extract was shown to contain the khat-specific alkaloids, cathinone and cathine (Figure 1), which also induced apoptosis in concentrations that were comparable to levels found in khat-exposed cells (Table 1). The morphological effects induced by khat were typical of the cell death previously reported in apoptotic cells (Kerr *et al*, 1972). The morphologically deformed cells were still able to restrict their influx of macromolecules (Figure 3), and cell death was, at least partially, dependent on *de novo* protein synthesis (Figure 6). Similar features have previously been reported in other models of apoptosis (Wyllie *et al*, 1980; Lanotte *et al*, 1991). Our results showed that khat had to be present for 0.5–0.75 h to be fully active (Figure 6). This suggested that khat-induced cell deaths were partly dependent on a commitment phase for induction of apoptosis as has also been reported previously during apoptotic cell death in acute myeloid leukaemia cells (Wyllie *et al*, 1980; Jensen *et al*, 1999). This is probably related to induction of specific genes during the cell death process although this is not a prerequisite for cell death by apoptosis (Wang and El-Deiry, 2003). Moreover, procaspase-3 was specifically activated by cleavage into 17 and 19 kDa protein fragments (Figure 4). This cleavage was inhibited by ZVAD-fmk, a pan-caspase inhibitor (Figure 8A). Activated caspase-3 is generally considered the main effector caspase during the execution phase of apoptotic cell death (Koester and Bolton, 1999; Herngartner, 2000; Vaux, 2002). That ZVAD-fmk, a pan-caspase inhibitor, could prevent the morphological effects of khat in submicromolar concentrations (Figure 8) further supported the observations above that khat-induced cell death was mediated by induction of apoptosis.

That the induction of cell death by khat was (1) synchronous, (2) occurred in most cells and (3) was very concentration dependent (Figure 3) suggests that the effect could be elicited through a specific mechanism(s). The mechanism(s) behind these observations remains enigmatic. Our results indicate that not only caspase-3 but also caspase-1 and -8 could be involved in the cascade of cellular events leading to khat-induced cell death. The caspase inhibitor ZIETD-fmk, which selectively inhibits caspase-8, counteracted the morphological effects of khat-induced apoptosis although with less potency than ZVAD-fmk (Figure 7). This could point to a role for the Fas/TNF receptor family of transmembrane death receptors in khat-induced cell death. Caspase-8 has a key position in the initiation of this death-receptor pathway by recruiting the procaspase-8 to the death-inducing signalling complex (Herngartner, 2000). However, its more specific role in khat-induced cell was not further tested in this study.

Also, the caspase inhibitor Z-YVAD-fmk, a potent inhibitor of caspase-1 (Schumann *et al*, 1998), inhibited khat-induced cell death (Figure 7). IL-1*β* is a known substrate for caspase-1 (Kuida *et al*, 1995). However, the role of caspase-1 in induction of apoptosis is controversial (Nicholson and Thornberry, 1997). Overexpression of caspase-1 has induced apoptosis in mammalian cells (Miura *et al*, 1993), whereas mice deficient in IL-1*β* develop normally, suggesting a less important role of caspase-1 in regulation of cell death during normal embryogenesis (Kuida *et al*, 1995). On the other hand, thymocytes from IL-1*β*-deficient mice were found to be resistant to *Fas*-induced cell death, showing an impairment of the normal regulation of apoptosis in these cells (Kuida *et al*, 1995). YVAD-fmk has protected HL-60 cells against camptothecin-induced apoptosis, but not the commitment to cell death suggesting a role for caspase-1 in the execution of apoptosis (Jensen *et al*, 1999). The observed 50% inhibition constant (IC_50_) of khat-induced apoptosis by Z-YVAD-fmk was 8 × 10^−7^ M as compared to 2 × 10^−8^ and 9 × 10^−8^ M, respectively, for Z-VAD and Z-IETD (Figure 8B). Knowing that the inhibition constant for caspase-1 by YVAD-CHO is several orders of magnitude lower than for inhibition of caspase-3 (Nicholson and Thornberry, 1997), and that experiments *in vitro* have shown IC_50_ for inhibition of IL-1*β* in the low *μ*M range (Kuida *et al*, 1995), our results *in vivo* thus could point to a role for caspase-1 in khat-induced apoptosis. It was not further tested whether that role could be related to the induction or the execution phase of apoptosis.

The main alkaloids present in khat leaves are cathinone, norpseudoephedrine (cathine) and norephedrine (Szendrei, 1980). These phenylpropylamines, structurally related to amphetamine and ephedrine, are considered the major bioactive compounds in khat extract (Kalix, 1992). To our knowledge, these khat-specific phenylpropylamines have not previously been reported to be associated with induction of apoptosis. Our own preliminary results indicate that cathinone induces a rather sensitive apoptosis in various human myeloid leukaemic cell lines. Amphetamine has been shown to induce apoptosis in fetal rat neocortical neurons (Stumm *et al*, 1999) and in PC12 cells (Oliveira *et al*, 2002) in a process generally considered to be associated with increased stress and formation of reactive oxygen substances. Moreover, the ephedrine-related catecholamine, norepinephrine, induces apoptosis in cardiac myocytes (Singh *et al*, 2001) by binding to *β*-adrenergic receptors through a mechanism that is dependent on activation of cyclic AMP-dependent protein kinase (Mann *et al*, 1992; Communal *et al*, 2000).

The potential mechanisms(s) for khat-induced apoptosis in human leukaemic cells is at present unknown. However, our results on khat-induced apoptosis point to an activation of the extrinsic cell death pathway. This is mainly based on the observation that cell death by khat (1) can be induced swiftly and synchronously in all cells, (2) is critically dependent on khat concentration, (3) is partly dependent on *de novo* protein synthesis, (4) is initially reversible upon removal of khat, (5) is sensitively blocked by a pan-caspase (ZVAD-fmk) inhibitor and by inhibitors selecting caspase-1 and 8. Our unpublished observations also suggest that the cell death process can also be rather sensitively modulated by tuning the expressed endogenous levels of Bcl-2 in leukaemic (IPC-81) cell lines.

## References

[bib1] Al-Ahdal MN, McGarry TJ, Hannan M (1988) Cytoxicity of khat *(Catha edulis)* extract on cultured mammalian cells: effects on macromolecule biosynthesis. Mutat Res 204: 317–322 2449610 10.1016/0165-1218(88)90105-x

[bib2] Al-Mamary M, Al-Habori M, Al-Aghbari AM, Baker MM (2002) Investigation into the toxicological effects of *Catha edulis* leaves: a short term study in animals. Phytother Res 16: 127–132 11933113 10.1002/ptr.835

[bib3] Al-Meshal I, Qureshi S, Ageel AM, Tariq M (1991) The toxicity of *Catha edulis* in mice. J Subst Abuse 3: 107–115 1687965 10.1016/s0899-3289(05)80011-2

[bib4] Al-Motarreb A, Baker K, Broadley KJ (2002) Khat: pharmacological and medical aspects and its social use in Yemen. Phytother Res 16: 403–413 12203257 10.1002/ptr.1106

[bib5] Al-Qirim TM, Shahwan M, Zaidi KR, Uddin Q, Banu N (2002) Effect of khat, its constituents and restraint stress on free radical metabolism of rats. J Ethnopharmacol 83: 245–250 12426093 10.1016/s0378-8741(02)00251-9

[bib37] Bøe R, Gjertsen BT, Doskeland SO, Vintermyr OK (1995) R8-Chloro-cAMP induces apoptotic cell death in a human mammary carcinoma cell (MCF-7) line. Br J Cancer 72: 1151–1159 7577461 10.1038/bjc.1995.479 PMC2033955

[bib6] Bruserud O, Hovland R, Wergeland L, Huang T, Gjertsen B (2003) Flt3-mediated signaling in human acute myelogenous leukemai (AML) blasts: a functional characterization of Flt3-ligand effects in AML cell populations with and without genetic Flt3 abnormalities. Haematologica 88: 416–428 12681969

[bib7] Carvalho F (2003) The toxilogical potential of khat. J Ethnopharmacol 87: 1–2 12787946 10.1016/s0378-8741(03)00100-4

[bib8] Communal C, Colucci WS, Singh K (2000) p38 mitogen-activated protein kinase pathway protects adult rat ventricular myocytes against beta-adrenergic receptor-stimulated apoptosis. Evidence for Gi-dependent activation. J Biol Chem 275: 19395–19400 10770956 10.1074/jbc.M910471199

[bib9] Dimba E, Gjertsen BT, Francis GW, Johannessen AC, Vintermyr OK (2003) *Catha edulis* (khat) induces cell death by apoptosis in leukemia cell lines. Ann NY Acad Sci 1010: 384–388 15033757 10.1196/annals.1299.070

[bib10] Gjertsen BT, Cressey LI, Ruchaud S, Houge G, Lanotte M, Døskeland SO (1994) Multiple apoptotic death types triggered through activation of separate pathways by cAMP and inhibitors of protein phosphatases in one (IPC leukemia) cell line. J Cell Sci 107: 3363–3377 7706392 10.1242/jcs.107.12.3363

[bib11] Herngartner MO (2000) The biochemistry of apoptosis. Nature 407: 770–776 11048727 10.1038/35037710

[bib12] Jensen PH, Cressey LI, Gjertsen BT, Madsen P, Mellgren G, Hokland P, Glieman J, Døskeland SO, Lanotte SO, Vintermyr OK (1994) Cleaved intracellular plasminogen activator inhibitor 2 in human myeloleukaemia cells is a marker of apoptosis. Brit J Cancer 70: 834–840 7947088 10.1038/bjc.1994.407 PMC2033559

[bib13] Jensen PH, Fladmark KE, Gjertsen BT, Vintermyr OK (1999) Caspase-1-related protease inhibition retards the execution of okadaic acid- and camptothecin-induced apoptosis and PAI-2 cleavage, but not commitment to cell death in HL-60 cells. Brit J Cancer 79: 1685–1691 10206278 10.1038/sj.bjc.6690269 PMC2362792

[bib14] Kalix P (1992) Cathinone, a natural amphetamine. Pharmacol Toxicol 70: 77–86 1508843 10.1111/j.1600-0773.1992.tb00434.x

[bib15] Kassie F, Darroudi F, Kundi M, Schulte-Hermann R, Knasmuller S (2001) Khat *(Catha edulis)* consumption causes genotoxic effects in humans. Int J Cancer 92: 329–332 11291066 10.1002/ijc.1195

[bib16] Kerr JFR, Wyllie AH, Currie AR (1972) Apoptosis: a basic biological phenomenon with wide-ranging implications in tissue kinetics. Brit J Cancer 26: 239–257 4561027 10.1038/bjc.1972.33 PMC2008650

[bib17] Kite GC, Ismail M, Simmonds M, Houghton P (2003) Use of doubly protonated molecules in the analyusis of cathedulins in crude extract of khat *(Catha edulis)* by liquid chromatography/serial mass spectrometry. Rapid Communicat Mass Spectrom 17: 1553–1564 10.1002/rcm.1085 12845580

[bib18] Koester SK, Bolton WE (1999) Differentiation and assessment of cell death. Clin Chem Lab Med 37: 311–317 10353477 10.1515/CCLM.1999.053

[bib19] Kuida K, Lippke JA, Ku G, Harding MW, Livingston DJ, Su MSS, Flavell RA (1995) Altered cytokine export and apoptosis in mice deficient in interleukin-1 converting enzyme. Nature 267: 1998–2000 10.1126/science.7535475 7535475

[bib20] Lanotte M, Riviere JB, Hermouet S, Houge G, Vintermyr OK, Gjertsen BT, Døskeland SO (1991) Programmed cell death (apoptosis) is induced rapidly and with positive cooperativity by activation of cyclic adenosine monophosphate-kinase I in a myeloid leukemia cell line. J Cell Physiol 146: 73–80 1846637 10.1002/jcp.1041460110

[bib21] Lee MM (1995) The identification of cathinone in khat *(Catha edulis)*: a time study. J Forens Sci 40: 116–121

[bib22] Mann DL, Kent RL, Parsons B, Cooper Gt (1992) Adrenergic effects on the biology of the adult mammalian cardiocyte. Circulation 85: 790–804 1370925 10.1161/01.cir.85.2.790

[bib23] Miura M, Zhu H, Rotello R, Hartweig ZA, Yuan J (1993) Induction of apoptosis in fibroblasts by IL-1 beta-converting enzyme, a mammalian homologue of the *C.elegans* cell death gene ced-3. Cell 75: 653–660 8242741 10.1016/0092-8674(93)90486-a

[bib25] Nicholson DW, Thornberry NA (1997) Caspases: killer proteases. TIBS 22: 299–306 9270303 10.1016/s0968-0004(97)01085-2

[bib26] Oliveira MT, Rego AC, Morgadinho MT, Macedo TRA, Oliveira CR (2002) Toxic effects of opioid and stimulant drugs on undifferentiated PC12 cells. Ann NY Acad Sci 965: 487–496 12105124 10.1111/j.1749-6632.2002.tb04190.x

[bib27] Rothman RB, Nga V, Partilla JS, Roth BL, Hufeisen SJ, Compton-Toth BA, Birkes J, Young R, Glennon RA (2003) *In-vitro* characterization of ephedrine-related stereoisomers at biogenic amine trasporters and the receptorome reveals selective actions and norepinephrine transporter substrates. J Pharmacol Exp Therapeut 307: 138–145 10.1124/jpet.103.053975 12954796

[bib28] Schumann RR, Belka C, Reuter D, Lamping N, Kirsching CJ, Weber JR, Pfeil D (1998) Lipopolysaccharide activates caspase-1 (interleukin-1-converting enzyme) in cultured monocytic and endothelial cells. Blood 15: 577–584 9427712

[bib29] Singh K, Xiao L, Remondino A, Sawyer DB, Colucci WS (2001) Adrenergic regulation of cardiac myocyte apoptosis. J Cell Physiol 189: 257–265 11748583 10.1002/jcp.10024

[bib30] Stumm G, Schlegel J, Schafer T, Wurz C, Mennel HD, Krieg JC, Vedder H (1999) Amphetamines induce apoptosis and regulation of bcl-x splice variants in neocortical neurons. FASEB J 13: 1065–1072 10336889 10.1096/fasebj.13.9.1065

[bib31] Szendrei K (1980) The chemistry of khat. Bull Narcotics 32: 5–35 6911031

[bib32] Toennes SW, Harder S, Schramm M, Niess C, Kauert GF (2003) Pharmacokinetics of cathinone, cathine and norephedrine after the chewing of khat leaves. Br J Clin Pharmacol 56: 125–130 12848785 10.1046/j.1365-2125.2003.01834.x PMC1884326

[bib33] Vaux DL (2002) Apoptosis and toxicology – what relevance? Toxicology 181: 3–7 12505277 10.1016/s0300-483x(02)00248-2

[bib34] Wang S, El-Deiry WS (2003) TRAIL and apoptosis induction by TNF-family death receptors. Oncogene 22: 8628–8633 14634624 10.1038/sj.onc.1207232

[bib35] Wyllie AH, Kerr JFR, Currie AR (1980) Cell death: the significance of apoptosis. Int Rev Cytol 68: 251–305 7014501 10.1016/s0074-7696(08)62312-8

[bib36] Zhou J, Ninghua T (2000) Application of a new TLC chemical method for detection of cyclopeptides in plants. Chin Sci Bull 45: 1825–1831

